# Are we adequately reviewing confusion inducing drugs in patients referred to the memory assessment service?

**DOI:** 10.1192/bjo.2021.520

**Published:** 2021-06-18

**Authors:** Siddhant Hegde, Rashi Negi, Hari Shanmugaratnam

**Affiliations:** 1 Queen's Hospital Burton, St Michaels Court; 2 St Michaels Court

## Abstract

**Aims:**

The aim of this quality improvement evaluation project is to establish the standard of current practice in relation to reviewing confusion inducing drugs (CIDs) at the time of referral, as it has been hypothesised that these medications contribute to short term cognitive impairment. This is essential in order to establish the validity of the diagnostic processes of dementia syndrome in the memory assessment services.

**Background:**

It has long been established that anti-cholinergic medications (ACMs) have contributed to short-term cognitive impairment in patients taking them. This is compounded with the fact that these medications may be continued without review, for longer than was originally intended. The impact of polypharmacy, subsequent anti-cholinergic burden, and the overlapping presence of delirium, may call into question the validity of a diagnosis of dementia in patients who have not been correctly vetted during the course of their assessment. This quality improvement evaluation aims to assess whether patients’ medications are being reviewed before diagnosing a memory disorder. This is in accordance with guidance set out by the NG97 NICE guidelines, The Royal College of Psychiatrists Memory Service National Accreditation Programme (MSNAP), and the National Institute on Ageing and Alzheimer's Association (NIA-AA).

**Method:**

All new referrals to the memory assessment service during July and August 2019 were systematically reviewed and data extracted from the memory referral document and entries on RIO from first point of contact. The following data were recorded: patient ID, GPCOG/6CIT score, final diagnosis, CID prescriptions and CID review.

**Result:**

The results were collated using a data-set of 216 patients (136 females and 80 males,) of which the mean age was 79 years. It was noted that 36% of patients had not had any sort of cognitive assessment before referral, which identifies an area for improvement. However the most substantial finding was that only 10 patients (5%) had a CID prescription review documented in the RIO notes.

**Conclusion:**

Our data suggest that in our memory assessment service, only a small proportion of patients are having a documented review of their CIDs prior to diagnosis of dementia. In order to improve this and thus improve compliance with guidelines from the Royal College of Psychiatrists MSNAP and the NIA-AA, measures will be taken to issue each dementia support worker and nurse with a CID prescription review card, which will list those medications to consider and flag for review.

